# Growth of water hyacinth biomass and its impact on the floristic composition of aquatic plants in a wetland ecosystem of the Brahmaputra floodplain of Assam, India

**DOI:** 10.7717/peerj.14811

**Published:** 2023-02-03

**Authors:** Durlov Lahon, Dhrubajyoti Sahariah, Jatan Debnath, Nityaranjan Nath, Gowhar Meraj, Majid Farooq, Shruti Kanga, Suraj Kumar Singh, Kesar Chand

**Affiliations:** 1 Department of Geography, Gauhati University, Guwahati, Assam, India; 2 Center for Climate Change and Water Research (C3WR), Suresh Gyan Vihar University, Jaipur, Rajasthan, India; 3 Center for Sustainable Development, Suresh Gyan Vihar University, Jaipur, Rajasthan, India; 4 GB Pant National Institute of Himalayan Environment (NIHE), Himachal Regional Centre, Kullu, Himachal Pradesh, India; 5 Current affiliation: Department of Geography, School of Environment and Earth Sciences, Central University of Punjab, VPO-Ghudda, Bathinda, India

**Keywords:** Wetland, Water hyacinth, Statistics, Biodiversity, Pollution, Spatial information sciences

## Abstract

Inland water plants, particularly those that thrive in shallow environments, are vital to the health of aquatic ecosystems. Water hyacinth is a typical example of inland species, an invasive aquatic plant that can drastically alter the natural plant community’s floral diversity. The present study aims to assess the impact of water hyacinth biomass on the floristic characteristics of aquatic plants in the Merbil wetland of the Brahmaputra floodplain, NE, India. Using a systematic sampling technique, data were collected from the field at regular intervals for one year (2021) to estimate monthly water hyacinth biomass. The total estimate of the wetland’s biomass was made using the Kriging interpolation technique. The Shannon-Wiener diversity index (*H*′), Simpson’s diversity index (*D*), dominance and evenness or equitability index (*E*), density, and frequency were used to estimate the floristic characteristics of aquatic plants in the wetland. The result shows that the highest biomass was recorded in September (408.1 tons/ha), while the lowest was recorded in March (38 tons/ha). The floristic composition of aquatic plants was significantly influenced by water hyacinth biomass. A total of forty-one plant species from 23 different families were found in this tiny freshwater marsh during the floristic survey. Out of the total, 25 species were emergent, 11 were floating leaves, and the remaining five were free-floating habitats. *Eichhornia crassipes* was the wetland’s most dominant plant. A negative correlation was observed between water hyacinth biomass and the Shannon (*H*) index, Simpson diversity index, and evenness. We observed that water hyacinths had changed the plant community structure of freshwater habitats in the study area. Water hyacinth’s rapid expansion blocked out sunlight, reducing the ecosystem’s productivity and ultimately leading to species loss. The study will help devise plans for the sustainable management of natural resources and provide helpful guidance for maintaining the short- to the medium-term ecological balance in similar wetlands.

## Introduction

Aquatic plants constitute a significant element of freshwater wetland communities in terms of ecosystem functioning, biomass production, species richness, and contribution to biodiversity (Wetzel, 2001). Recently, it has acquired greater attention due to its potential use in wastewater treatment (Huynh, Chen & Tran, 2021; Prasad et al., 2021) and food sources. Moreover, it is essential for a freshwater ecosystem in general due to its action in providing habitat, and food for many wild animals, such as waterfowl and fish. It absorbs nutrients that are dissolving in the water, regulates the nutrient cycle, and greatly impacts oxygen availability in the water. Furthermore, the aquatic plant is used for environmental assessment and water quality monitoring (Schneider & Melzer, 2004), and hence the absence or presence of particular plant species in a water body can show good or bad water status (Sugier & Lorens, 2010). But some invasive aquatic plants, especially water hyacinth, adversely affect the ecological health of the freshwater ecosystem due to their nature of rapid growth. It is a free-floating perennial plant that is considered one of the worst nuisance aquatic plants (Holm et al., 1977; Eid & Shaltout, 2017) and has received worldwide attention due to its fast spread and crowded growth (Rezania et al., 2015). It produces a significant amount of aquatic biomass and can completely cover natural water bodies, reducing oxygen levels for fish, displacing native aquatic species, creating an ideal habitat for disease-carrying mosquitoes (Stohlgren et al., 2013), and obstructing sunlight which results in a serious impact on various lifeforms in the ecosystem (Rumjit et al., 2022). Moreover, the large infestation of water hyacinth can prevent navigation, fishing, river transport, and boating and can clog dams. It can reproduce quickly *via* both vegetative and sexual ways, resulting in a high rate of biomass production in a short period (Penfound & Earle, 1948; Fernando, 2003). It is native to Brazil and is considered one of the most prolific colonizers among the plant communities that were introduced to India as an ornamental plant in West Bengal in the early twenty century (Bote, Naik & Jagadeeshgouda, 2020). The average height of a water hyacinth is about 40 cm, but it can get as tall as 1 m under exceptional circumstances. Water hyacinth has glossy, dark green leaves that are broad and thick. At 2.54 cm below the surface of the water, the roots have a deep purple-black color and take the form of dangling, elongated treads (Dersseh et al., 2019). The plant cannot stand alone and is always seen in colonies. It can double its size within five to fifteen days (Dersseh et al., 2019), and under favorable conditions, it can reach up to 17.5 metric tons per hectare per day (Kunatsa et al., 2013). Water hyacinth reproduces *via* both sexual and asexual methods. In sexual reproduction, new plants are grown through seeds, whereas asexual reproduction is by budding through vegetative reproduction systems (Dersseh et al., 2019). According to the research of Penfound & Earle (1948), just ten parent plants can produce 655,360 offspring in one growing season. Another study by Vymazal (2008) found that just ten plants could cover 0.4 hectares of freshwater with a harvest of 600,000 throughout an 8-month growing season. Water hyacinths can also propagate sexually, expanding their population by the spread of flower seeds. However, the climate and the season have a role in this production system. The seed can be spread in several ways, such as by humans, birds, floods, etc. Individual plants are usually separated from the mother plant, spread by wind and water currents, and can create a new mat on the water’s surface. The water surface covered by the hyacinth mat doubles itself within 4 to 7 days only (Hill & Coetzee, 2008; Dersseh et al., 2019). This plant grows in a wide range of temperatures between 13° to 40 °C but optimally grows from 25° to 30 °C (Wilson et al., 2001). Thus, tropical as well as subtropical regions in the world are favorable for the growth of water hyacinths. Since India has experienced both tropical and subtropical climatic conditions, it is also suitable for the growth and spread of this invasive aquatic weed.

An overabundance of water hyacinths has recently significantly impacted numerous freshwater lakes across the world (Tobias et al., 2019). Rapidly expanding from the Amazon, it can now be found throughout South America, Africa, the Caribbean, and Southeast Asia (Dersseh et al., 2019; Navarro & George, 2000). This plant has caused significant environmental and socioeconomic concerns in Lake Victoria, the world’s second-largest lake (Williams, Duthie & Hecky, 2005). Since 2011, water hyacinth has been invading Lake Tana, Ethiopia. Currently, the expansion rate of water hyacinth is too high, necessitating collaboration among all stakeholders to bring it under control (Dersseh et al., 2019). Water hyacinth infestations are an issue in many Southeast Asian nations, including India, China, Thailand, Vietnam, Laos, and Indonesia (Charudattan et al., 1995).

Wetlands, or floodplains, are low-lying areas bordering rivers that are seasonally inundated by the runoff from the main river channel. Wetlands cover about 10 percent of the earth’s surface, and 15 percent are floodplain wetlands (Mukherjee & Pal, 2021). The ecological benefits of floodplain wetlands are very significant (Meraj, 2020; Meraj et al., 2021; Meraj et al., 2022). For example, they can reduce carbon levels, prevent flood damage, provide a home for a variety of threatened plants and animals, preserve groundwater storage, and naturally clean sewage (Bhat et al., 2013; Bhat et al., 2014; Waltham et al., 2019). According to Costanza et al. (1997), floodplain wetlands and inland swamps are far more beneficial to the ecosystem than rivers, forests, grasslands, and lakes. Assam, a central, northeastern state of India, contains vast floodplain wetlands with a rich diversity of flora and fauna. Typical ox-bow lakes, meanders, back swamps, residual channels, and sloughs can be found in the Brahmaputra river valley of this state; however, it is frequently difficult to verify their identity due to natural and manmade alterations (Sugunan & Bhattacharjya, 2000). The frequently shifting courses of rivers and their tributaries in the upper portions are responsible for the prevalence of these floodplain wetlands in the Brahmaputra basin. The heavy discharge of water triggers the process of meander cut-offs that leads to the formation of ox-bow lakes. The river passes through the zone of intense seismic activity, and hence, different tectonic depressions were formed due to earthquakes. Moreover, the Brahmaputra is prone to frequent and heavy floods, which break the levees and form back swamps and sloughs (Sugunan & Bhattacharjya, 2000). These floodplain wetlands of the Brahmaputra basin have a rich diversity of flora and fauna. But the excessive growth of some invasive free-floating aquatic plants, especially water hyacinth, creates various problems in these wetlands like navigation, fishing, boating tourism, deterioration of water quality, and reduced species diversity. Merbil, Deepor Beel, the lowland area of Dibru-Saikhowa National Park, the Maguri Motapung wetland, the wetland area of Kaziranga National Park, and the Pani Dihing Wild Life Sanctuary are just some of the important wetlands in the Assam state where the government has spent a substantial amount of money to eradicate this aquatic weed. The thick cover of water hyacinth decline the productivity of the ecosystem as a result of its inability to obtain light for photosynthesis, which led to lower species diversity in local and regional level.

The primary objective of the current study was to estimate the biomass of water hyacinths in the freshwater wetlands of Merbil and determine how it might affect the floristic composition of aquatic plants. Several studies have been conducted regarding the biomass estimation of water hyacinth in different freshwater bodies worldwide to understand its impact on aquatic systems. However, these studies have not thoroughly examined how water hyacinth biomass affects the floristic compositions of other aquatic plants. In this context, the current study was carried out to evaluate the growth of water hyacinth biomass and investigate its impact on the floristic composition and species diversity of aquatic macrophytes, which was unusual in previous research. The study will aid in developing natural resource sustainability management plans and provide useful guidelines for preserving the local ecological balance in small wetlands over the short to medium term.

## Material and Methods

### Study area

**Figure 1:**
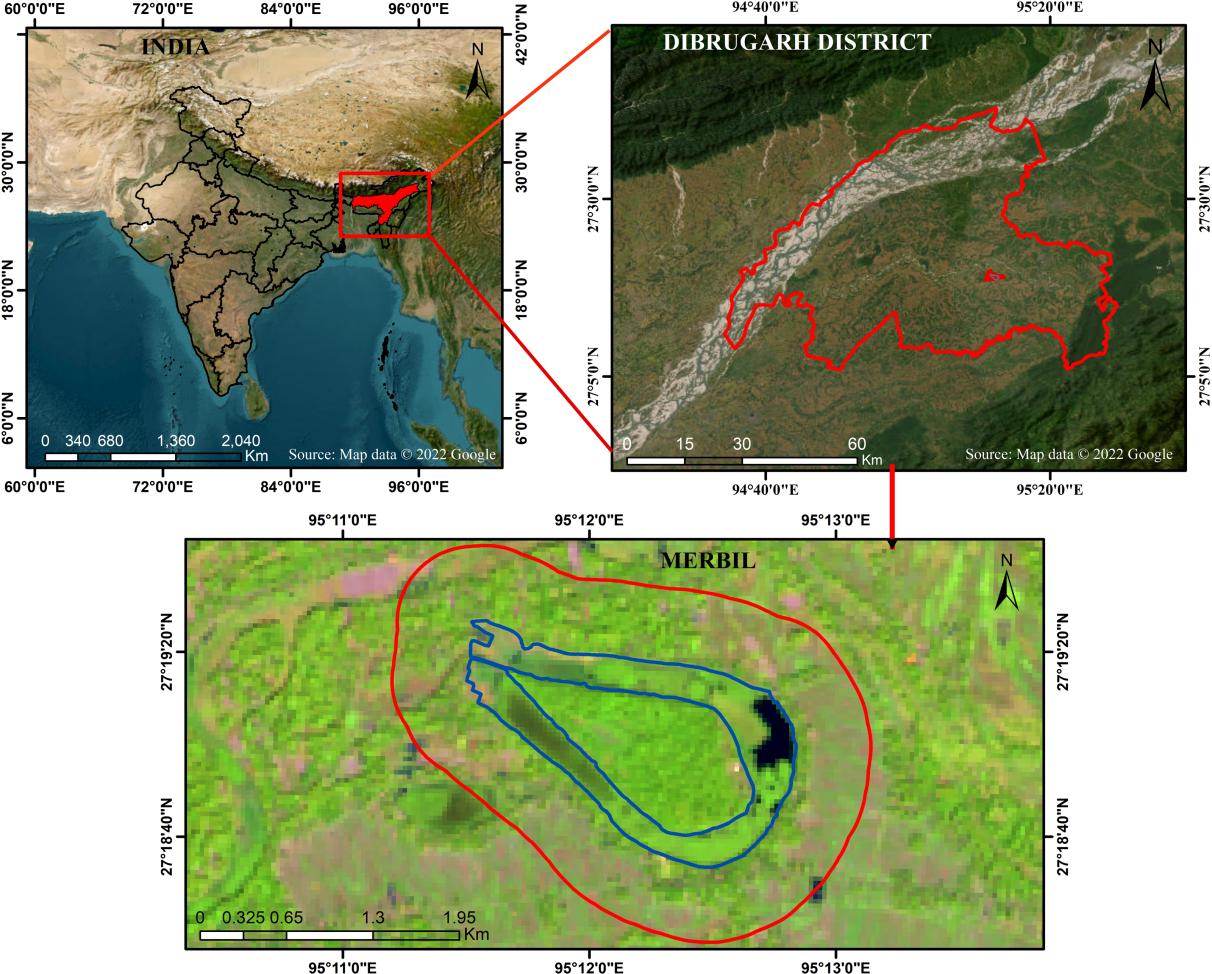
Location map of the Merbil wetland. Map data: (c) 2022 Google.

The Merbil is a tiny freshwater lake in the North East India biogeographic zone, located in the upper Brahmaputra flood plain of Assam, India. The Burhi Dihing River, a major south-bank tributary of the Brahmaputra, has meandered, creating a lake that resembles an ox-bow. It stretches from 95°11′00″E to 95°13′00″E longitudinally and 27°17′30″N to 27°20′00″N latitudinally (Fig. 1). The Burhi Dihing River runs north and west, the Charaideo district lies to the south and Dihing Patkai National Park to the east. According to Koppen’s (Oliver & Fairbridge, 1987) classification system, the region’s climate is sub-tropical humid and regulated by the region’s distinctive topographical features and monsoonal circulation (Kumar & Dimri, 2018; Debnath et al., 2022a; Debnath et al., 2022b). May marks the beginning of the research site’s rainy season, which lasts through October. Pre-monsoon showers begin in the middle of April, but the actual monsoon rains do not begin until May (Datta & Singh, 2004). This freshwater lake is home to a wide range of flora and fauna due to its favorable climate and the lake’s proximity to a variety of different environments. Numerous waterfowl and other avian species, both migratory and permanent, call this wetland home. In 2010, the ‘Sasoni Merbil Eco-Tourism Project’ started to preserve the biodiversity of the wetland. Various aquatic plants are grown in the wetland, including free-floating, floating-leaved, emergent, and submerged plants. The water hyacinth (*Eichhornia crassipes*), which has favorable climatic conditions for growth, is one of the lake’s most common and densely populated aquatic plants. In addition to impacting the lake’s ecological health, the rapidly increasing water hyacinth (*Eichhornia crassipes*) also affects the ecosystem services, recreational opportunities, and floral features of the lake. As a result, this lake has been selected as a suitable site for determining the biomass of water hyacinths and how it affects the floristic composition of aquatic plants. A further benefit of calculating water hyacinth biomass (*Eichhornia crassipes*) and evaluating its influence on floristic composition is that the wetland is not significantly affected by either natural or human-caused disturbances. The lake’s small size also helps to increase the study’s accuracy and reduce the cost and time.

### Collection of data

**Figure 2:**
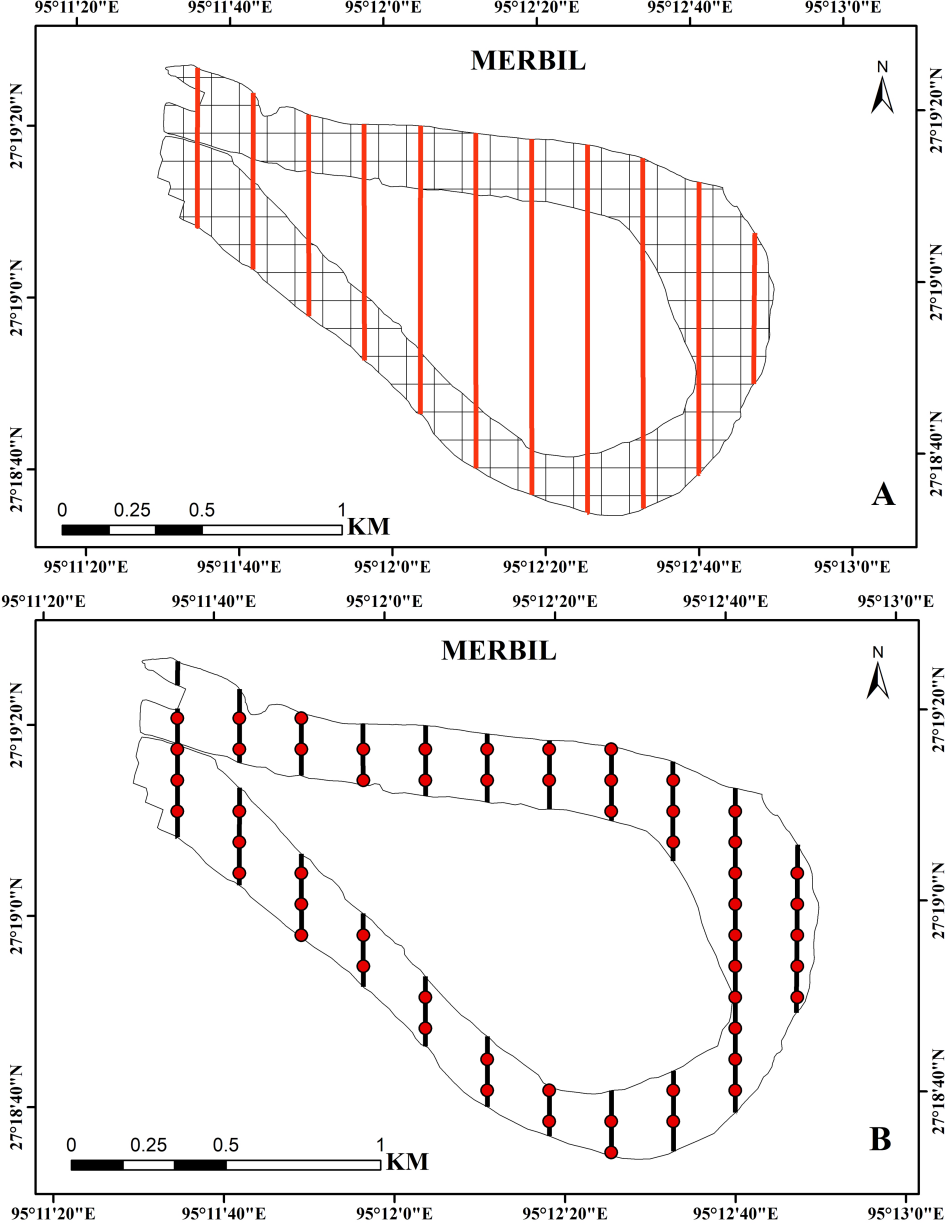
(A) Study site with 100 × 100 m grids and evenly distributed transect line, (B) regular interval points on the transect line used for the sample collection at the Merbil wetland.

Using the systematic sampling technique, the plant samples were collected at regular intervals during the study year (2021). This sampling technique helps to achieve more efficient results due to its uniform coverage of the study area. In the present study, the freshwater wetland was divided into 178 square grids of 100 × 100 m using the ‘Fishnet’ function of ArcGIS (Madsen & Wersal, 2017; Bordoloi et al., 2020), and then several evenly spaced transect lines were drawn on the study site with an interval of 200 m (Fig. 2). Aquatic plants of waterbodies can be sampled using a variety of techniques like surface inventory, driver inventory, surface mapping, remote sensing, and line transect, depending on the degree of precision and detail needed to meet the study’s objectives (Parsons, 2001). Here, we used transect line sampling because the evenly spaced transects help achieve a more uniform representation of different areas within the sampling site (Titus, 1993a; Titus, 1993b). A total of 55 sampling points were placed along these transect lines with a predetermined spacing, and collected samples from all these points were through a small boat. The line transect method was used to collect samples because it allows for the collection of presence/absence data, cover data, and density and abundance measurements by using quadrants along transects (Greig-Smith, 1983; Titus, 1993a; Titus, 1993b; Getsinger et al., 1997). The line transect technique is specifically beneficial for characterizing aquatic plant groups on small study sites over time and assessing management effectiveness in small plots (Madsen & Wersal, 2017). The sampling points covered only about 30% of the total grids because it is costly, time-consuming, and risky to collect a large number of sample points in the aquatic environment. Each sampling location was divided into a 1 m^2^ quadrant, and its coordinates were captured using a global positioning system (GPS).

The aquatic plant samples regarding the biomass estimation were collected from the direct biomass sampling technique (Sanna et al., 2010) or the direct harvesting method (Grace, 1974; Downing & Anderson, 1985). It is emphasized that direct sampling methods always achieve more accuracy than any other allometric method (Westlake, Spence & Clarke, 1986). Biomass samples can be collected either by destructive methods, where plants are collected from the site and weighed, or by non-destructive methods, where an alternate measure related to weight, such as height, length, width, etc., are calibrated using sub-sampling destructive plant samples measuring weight and the two quantitative variables related by regression analysis (Madsen, 1993). But in both methods, some destructive biomass samples are needed. A partial sampling technique, known as the destructive sampling technique, was used in the current study to collect plant samples without destroying the entire population (Sanna et al., 2010). Subsequently, the total number of individuals was counted, and 20 percent of the total number of individuals in the quadrant was clipped as samples.

### Sample processing and estimation of biomass

Sample processing can be done in three major processes: biomass sample separation and cleaning, drying and weighing. The target sample was separated and washed properly in the field to remove debris materials. After properly cleaning the samples, it weighed to obtain the average fresh weight biomass or the amount of green biomass (GM) of each quadrant’s individual stem of water hyacinth (Delina, Dayawansa & Silva, 2019; Madsen & Madsen, 1993). The value of average biomass per individual was multiplied by the total number of plants of the particular quadrant to obtain the total biomass. The fresh weight was calculated immediately after collection and washing because decomposition may take place if it was not weighed immediately. The average biomass was determined using the formula given by Sanna et al. (2010).

(1) \begin{eqnarray*}Bi \left( q \right) = \frac{Bp \left( q \right) }{I \left( q \right) } .\end{eqnarray*}

Where *B*_*i*(*q*)_ is the individual’s biomass of quadrant, *I*_(*q*)_ is the number of plants harvested for biomass in the partial sample in quadrant *q*, and *B*_*p*(*q*)_ is the measured biomass of the partial sample in quadrant *q*.

### Total biomass estimation

Spatial interpolation was used to estimate the overall wetland biomass. It is a mathematical technique for making predictions about the location of things for which we just have partial information. Some popular interpolation methods include kriging and inverse distance weighted (IDW) interpolation. We used the kriging interpolation method in the current study because it is founded on statistical models incorporating autocorrelation, which have been reported to provide promising results using the dataset used in this study (Oliver & Webster, 2007). In addition, to generate a prediction surface, kriging also provides some measure of the certainty or accuracy of the predictions, unlike the IDW method (Burrough, 1986; Li et al., 2020; Munyati & Sinthumule, 2021). These include the methods of ordinary kriging, simple kriging, indicator kriging, universal kriging, and probability kriging interpolation (Xiao et al., 2016; Su et al., 2020). It is an ideal estimating method that does not favor any one location over another, making it suitable for defining regionally-specific variables such as the above-ground biomass of forest and aquatic plants (Burrough, 1986). It’s possible to get several different kinds of output surfaces from kriging, such as predictions, prediction standard error, and probability distributions. Thus, kriging is a very adaptable interpolator to well-distributed data with no discontinuity (Ohmer et al., 2017). The following formula was applied to this method to estimate water hyacinth biomass at the current study site.

(2) \begin{eqnarray*}Z \left( So \right) =\sum _{i=1}^{n}\rightthreetimes iZ \left( Si \right) .\end{eqnarray*}

*Z*(*Si*) is the value measured at site Ith location. ⋌*i* is a weight associated with a location I that is currently unknown, is the location at which predictions will be made, and *n* is the total number of locations for which values have been measured. The importance of ⋌*i* in kriging is determined by the model that is fit to the data, the distance to the predicted position, and the spatial correlations between the measured values in the area around the predicted site.

### Semivariogram model

**Figure 3:**
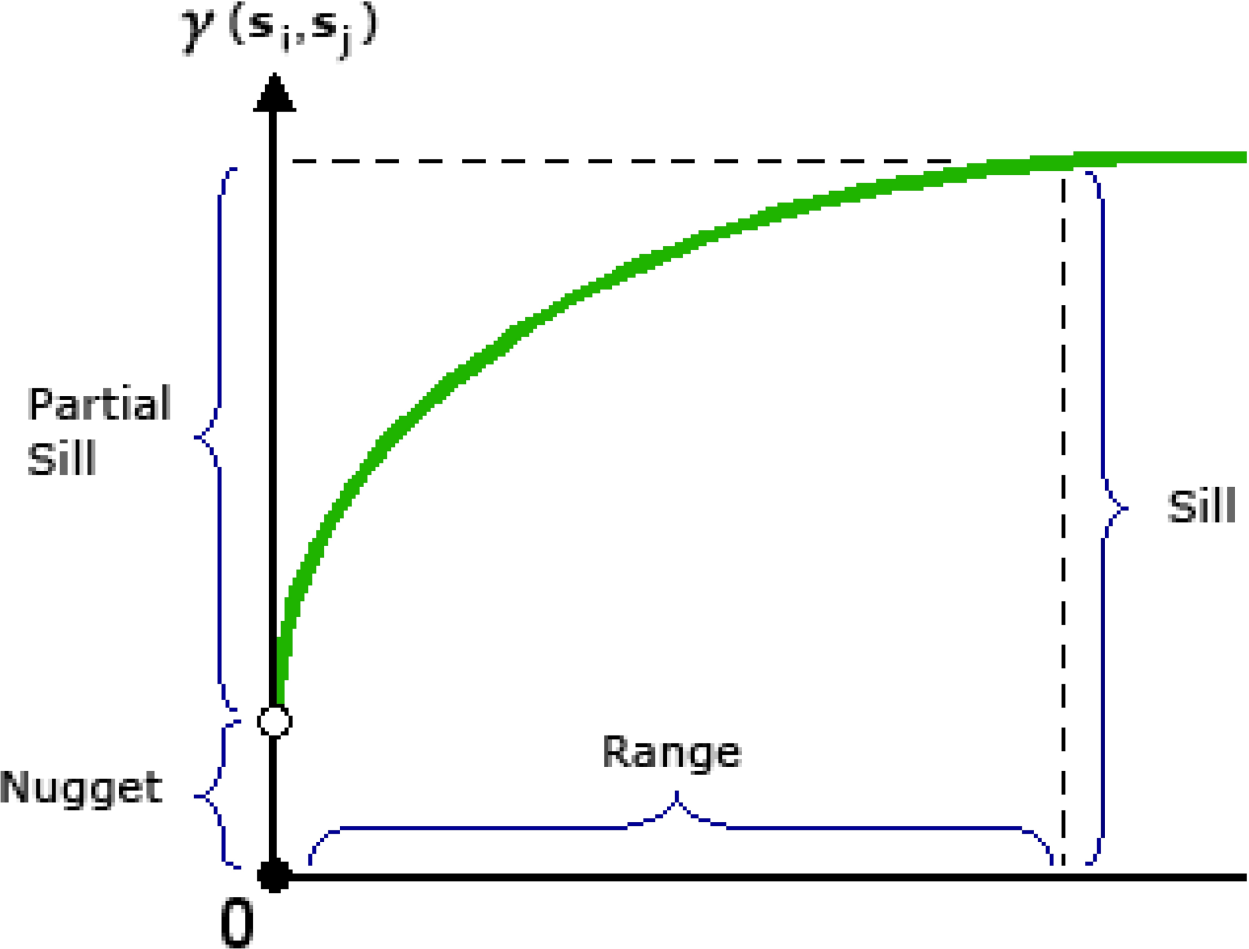
Typical semivariogram and its components.

There are different methods, like the semivariogram and Moran’s I, to measure the spatial autocorrelation between the variables. In the present study, the semivariogram model has been used to measure the average decline in the similarity between two random variables as their distance increases. It is one of the most suitable statistical techniques to indicate spatial correlation and evaluation of the variance of attributes with the distance between all pairs of sample locations (Liu, Xie & Xia, 2013). Due to its ability to reveal the spatial autocorrelation of datasets, semivariogram modeling is a crucial part of spatial description and prediction. Empirical semivariograms are typically modeled using linear, spherical, exponential, or Gaussian functions. Once the data dependence or autocorrelation has been defined, the fitted semivariogram model can be utilized to produce a forecast. Models are typically described using the range, sill, and nugget characteristics (Fig. 3) (Stein, 1999; Li et al., 2020). The range is the separation between when the model flattens out and when the data stop being correlated. Locations within the sample that are closer together than the range apart exhibit spatial autocorrelation, while locations further apart do not. With a value of sill, the semivariogram model reaches its maximum dynamic range. It is a measure of the dispersion in the data. Nugget refers to the point on the *y*-axis where the semivariogram model makes a right angle. If the y-intercept of the semivariogram model is 2, for instance, then 2 is the nugget. At a distance of zero, the semivariogram should have a value of 0. Semivariograms typically display a nugget effect when a value greater than zero is seen at infinitesimally small separation distances (Arc-GIS Desktop). One way to express the level of spatial autocorrelation of a localized variable is through the nugget effect or the ratio between the nugget and the sill (Curran, 1988).

The nugget effect can be considered a bulk parameter in the variogram model function that includes the local roughness of the variable under investigation and the uncertainty brought on by the discrete sampling pattern. It can as well include incorrect analytical data (Hofmann, Darsow & Schafmeister, 2010). As the nugget effect increases, both the spatial variability and spatial autocorrelation increase. Suppose the nugget effect is <0.25, it is considered strong spatial dependence or autocorrelation, while if the nugget effect ranges between 0.25–0.75, it is considered moderate spatial dependence, and nugget effect >0.75 denotes little spatial autocorrelation or weak spatial dependency (Xiao et al., 2016; Li et al., 2020).

### Validation

Kriging’s cross-validation prediction accuracy can be calculated as the root-mean-squared error (RMSE), mean-error (ME), mean-standardized error (MSE), root-mean-squared standardized error (RMSSE), and average standard error (ASE) (Li et al., 2020).

(3) \begin{eqnarray*}& & \mathrm{ME}= \frac{\sum _{i=1}^{n}(Yi-Xi)}{n} \end{eqnarray*}

(4) \begin{eqnarray*}& & \mathrm{RMSE}=\sqrt{\sum _{i=1}^{n}{ \left( \frac{Yi-Xi}{\tilde {O}i} \right) }^{2}/n}\end{eqnarray*}

(5) \begin{eqnarray*}& & \mathrm{MSE}= \frac{\sum _{i=1}^{n} \frac{ \left( Xi-Yi \right) }{\tilde {O}i} }{n} \end{eqnarray*}

(6) \begin{eqnarray*}& & \mathrm{RMSSE}=\sqrt{\sum _{i=1}^{n}{ \left( \frac{Xi-Yi}{\tilde {O}} \right) }^{2}/n}.\end{eqnarray*}

Where *Yi* represents the observed values, *Xi* represents the predicted values, $\tilde {O}i$ is the standard error of predicted values, and *n* is the number of samples used.

### Floristic analysis

Floristic composition, also termed plant species composition, generally refers to the specific characteristics of the plant species present in a certain geographical area. Understanding forest ecology and ecosystem processes require an in-depth examination of floristic composition, which is essential for forest management (Addi et al., 2016; Sewale & Mammo, 2022). The study of the floristic composition of aquatic plants is useful to assess the health of freshwater ecology and help safeguard threatened species to maintain the balance of the ecosystem. In the present study, the density and frequency were calculated using the formula recommended by Maszura et al. (2018) and Hailu (2017).

(7) \begin{eqnarray*}& & \text{Density}= \frac{\text{Total no. of individuals of a species in all the sampling units}}{\text{Total no. of sampling units studied}} \end{eqnarray*}

(8) \begin{eqnarray*}& & \text{Relative Density}= \frac{\text{No. of individuals of the species in all quadrantes}}{\text{No. of individuals of all species in all quadrantes}} \times 100\end{eqnarray*}

(9) \begin{eqnarray*}& & \text{Frequency}= \frac{\text{No. of quadrantes in which the species occurred}}{\text{The total No. of quadrant studied}} \times 100\end{eqnarray*}

(10) \begin{eqnarray*}& & \text{Relative Frequency}= \frac{\text{Frequency value of an individual species}}{\text{Total frequency values of all species}} \times 100.\end{eqnarray*}

Shannon-Wiener Diversity Index (*H*′), Simpson’s diversity index (*D*), and Dominance and Evenness or Equitability Index (*E*) were used to estimate the diversity of aquatic species in the wetland. Dominance was calculated using the following formula (unbiased):

(11) \begin{eqnarray*}D= \frac{\sum _{i}ni(ni-1)}{n(n-1)} .\end{eqnarray*}

Where *ni* is the number of individuals of taxon *i*. varies from zero (total absence) to one (1) (one taxon dominates the community completely). The following formula was used to determine Simpson diversity, where pi represents the percentage of people. The Simpson index, which evaluates community evenness, typically falls between 0 and 1.

(12) \begin{eqnarray*}D=1-\sum p{i}^{2}.\end{eqnarray*}

Using the following equation (Sewale & Mammo, 2022; Meragiaw et al., 2021), we were able to determine Shannon’s Equitability or Evenness as a function of the ratio of observed diversity to maximal diversity.

(13) \begin{eqnarray*}J= \frac{- \sum \begin{array}{@{}c@{}} \displaystyle s\\ \displaystyle i=1 \end{array}Pi\dot {\mathrm{ln}}Pi}{\mathrm{ln}S} = \frac{{H}^{{^{\prime}}}}{\mathrm{ln}S} .\end{eqnarray*}

*H*′ max = ln *S*, where ln is the natural logarithm, *S* is the total number of species in the sample, and *J* is the evenness. Parity or fairness is measured on a scale from 0 to 1. When the evenness index is high, species are distributed uniformly over a landscape.

Shannon-Wiener diversity index (*H*′) was calculated as:

(14) \begin{eqnarray*}{H}^{{^{\prime}}}=-\sum pi\ln \nolimits pi.\end{eqnarray*}

Where *pi* is the proportion of individuals, and *n* is the total number of individuals. Average Shannon diversity index values fall between 1.5 and 3.5, with 4.5 being an extreme outlier (Ifo et al., 2016).

## Results

### Analysis of semivariogram parameters

**Figure 4:**
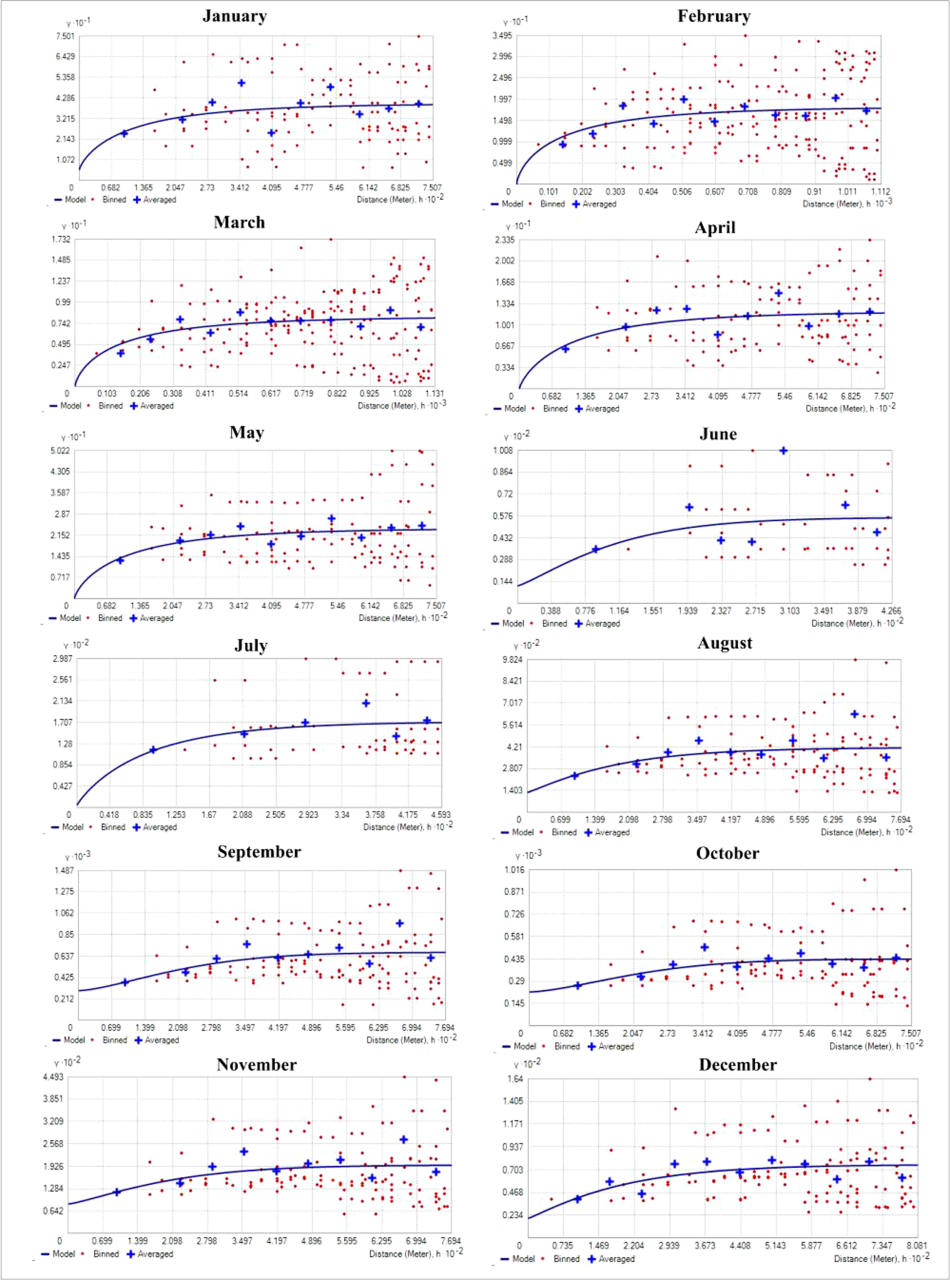
Empirical semivariogram model for different month (the *y* axis represents value of semivariogram and *x* axis represent distance).

Table 1 shows the result of semivariogram parameters used in spatial predictions for variables. During the months of January and March, the matured water hyacinth started to dry and decompose due to low rainfall as well as the decreasing water level of the wetland. Most of the parts become dry, and human influence increases continuously on this wetland. On the other hand, from March, the pre-monsoon started in the area, and again the water hyacinth started to reproduce gradually through vegetative and sexual methods. But during this period (January and July), the rate of biomass production of water hyacinth was low as compared to the second half of the year. Due to low biomass, the spatial variability of water hyacinth biomass was also small, and hence the nugget effect of water hyacinth biomass was <0.25 in these months, which means strong spatial autocorrelation. With increasing temperatures, rainfall, and water levels, water hyacinth biomass has been increasing in most parts of the wetland since July. As the spatial heterogeneity in biomass between the surface of the no-water hyacinth and the rest of the wetland increased, the nugget effect was observed to have a range between 0.25 and 0.75, showing moderate spatial dependence (Fig. 4).

**Table 1:** Result of semivariogram parameters used in spatial predictions for variable.

**Month**	**Range**	**Sill**	**Partial sill**	**Nugget**	**Nugget effect**
January	500.46	39.99	34.47	5.52	0.14
February	741.47	18.19	18.19	0	0
March	753.71	8.19	8.19	0	0
April	500.46	12.07	12.07	0	0
May	500.46	23.84	23.84	0	0
June	284.42	56.5	45.08	11.42	0.20
July	306.18	172.42	167.84	4.58	0.02
Augusta	512.91	415.88	288.84	127.04	0.30
September	512.91	674.37	379.89	294.48	0.43
October	500.46	435.13	214.62	220.51	0.50
November	512.91	196.03	111.84	84.19	0.42
December	538.74	75.52	55.38	20.14	0.26

### Estimation of water hyacinth biomass

**Figure 5:**
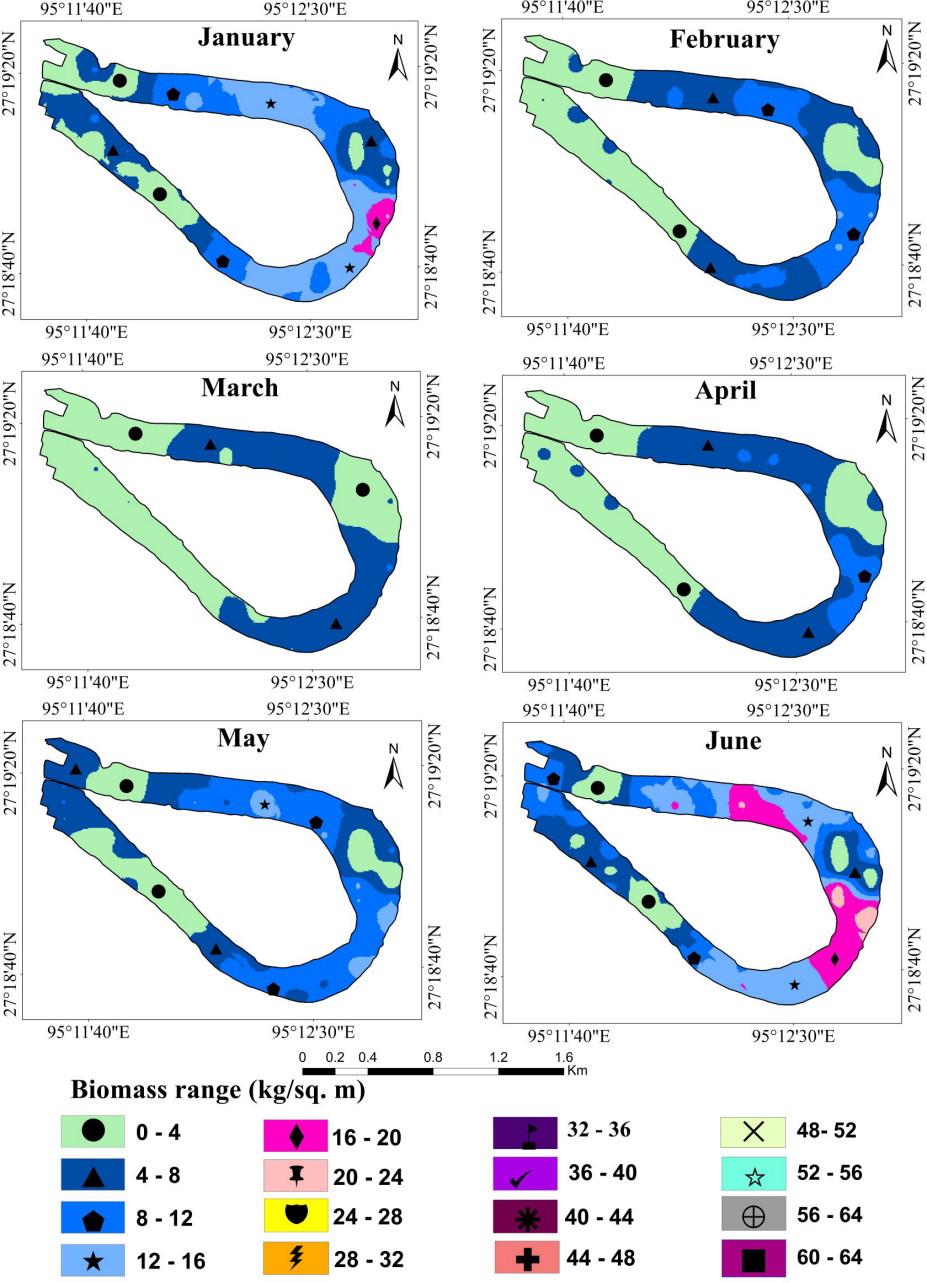
Fresh biomass of wetland for the month of January to June, 2021.

Wetland biomass estimation plays a crucial role in understanding the dynamic changes in the wetland ecosystem (Liao, Shen & Dong, 2013; Du et al., 2017). Table 2 shows the average predicted and total biomass (kg/m^2^) of water hyacinth over one year. The kriging interpolation method was used to calculate the average predicted biomass. The results suggest that the biomass trend of water hyacinth in the Merbil wetland between January and March was negatively growing. Minimum plant biomass was observed in March, only 3.80 kg/m^2^ or 38 t/ha (Fig. 5).

The study area received very little rain during this time, which reduced the wetland’s water level. It was also observed that the January and February weather is not ideal for the rapid growth of water hyacinth plants in the Merbil wetland. Furthermore, human intervention was at its peak during this month. The western section of the wetland has dried up and was used as grazing land. Although a large portion of the wetland produced a large amount of biomass, during the dry season, biomass production decreased significantly. As a result, average biomass production decreased.

**Figure 6:**
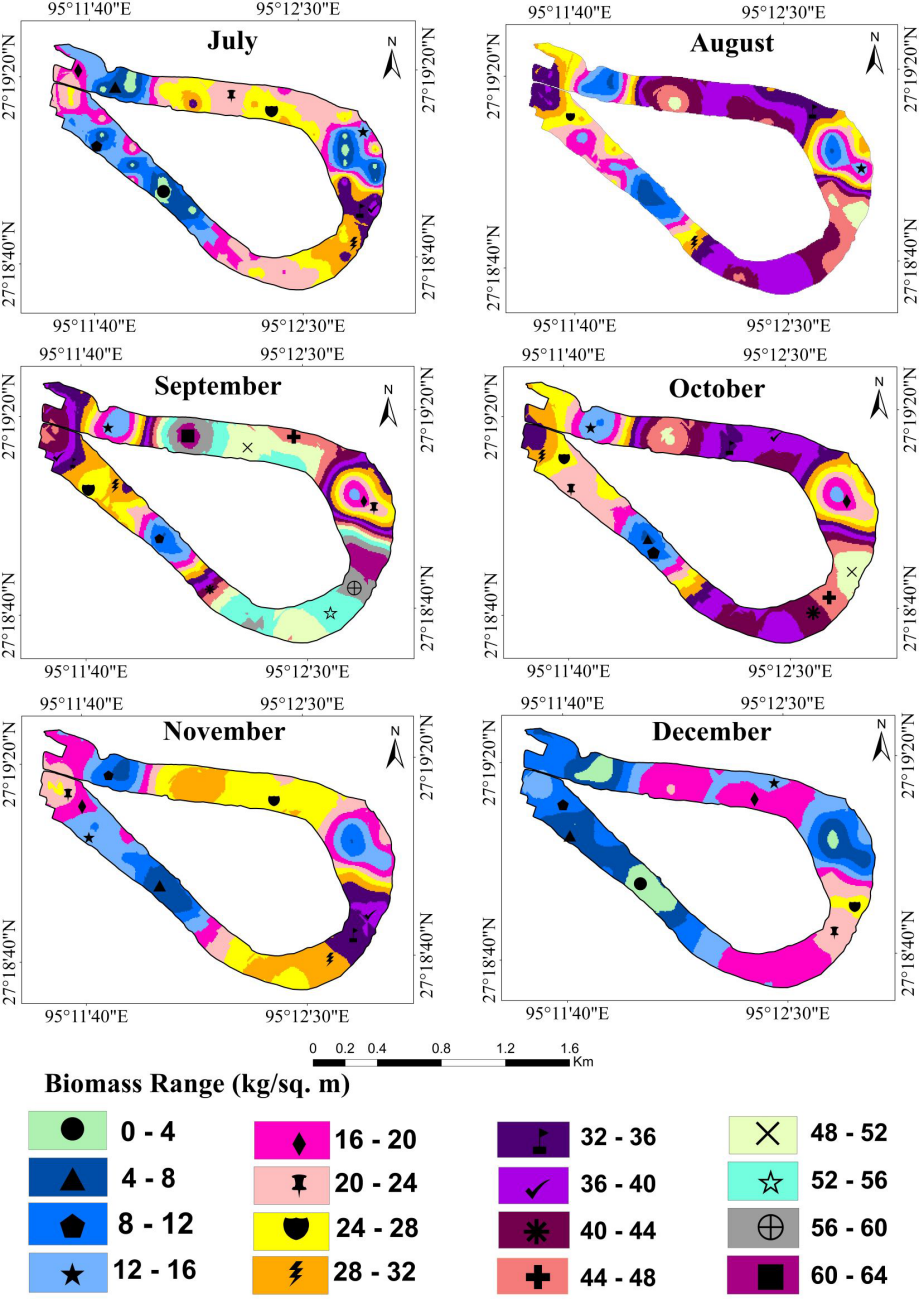
Fresh biomass of wetland for the month of July to December, 2021.

The result shows that from April onwards until September, water hyacinth biomass increased steadily with an increase in temperature and rainfall. Due to favorable growth conditions, maximum biomass of 40.81 kg/m^2^ or 408.1 t/ha was observed in September (Fig. 6). Under ideal conditions, its biomass can reach up to 400 tons/ha (Dersseh et al., 2019). The growth was visible in both vertical and horizontal directions during the first three months (April-June), but only in the vertical direction after that. The number of plants per square meter quadrant area increased rapidly from April to June, and then the number of plants retained in the system was more or less similar until September. This may have been due to the high density, which inhibited the growth of plants. During this period, the temperature, rainfall, and humidity are optimal for growing water hyacinth plants. Again, from October to December, biomass growth was observed to tend to decrease. In October, almost 22 percent of biomass had declined from the previous month. Meanwhile, rainfall became scarce, and the temperature started to drop from October onwards. Due to low rainfall, the water level of the wetland was decreased at the study site.

**Table 2:** Month wise biomass statistics of the study site.

**Month**	**Average predicted biomass (kg/m^2^)**	**Total biomass (in tons)**	**Biomass per hectors (in tons)**
January	8.77	9,997.8	87.7
February	5.29	6,030.6	52.9
March	3.8	4,332	38
April	4.54	5,175.6	45.4
May	6.88	7,843.2	68.8
June	10.81	12,323.4	108.1
July	16.38	18,673.2	163.8
Augusta	30.75	35,055	307.5
September	40.81	46,523.4	408.1
October	31.77	36,217.8	317.7
November	21.56	24,578.4	215.6
December	12.86	14,660.4	128.6

In the Merbil wetland, the highest 46523.4 tons of biomass were produced in September, while the lowest 4332 tons were produced in March. This huge amount of water hyacinth biomass significantly impacts the ecology of the lake. According to Lorber, JW & Reddy (1984), Henry-Silva, Camargo & Pezzato (2008) and Dersseh et al. (2019), water hyacinths have produced an enormous amount of biomass in an aquatic ecosystem.

### Evaluation of model accuracy

The ME shows how skewed the predictions are and needs to be near zero for fair techniques (Oliver & Webster, 2007). The root-mean-squared error (RMSE) reveals how near the predicted values are to the actual ones and serves as a measure of average forecast precision. A lower margin of error suggests more confidence in the prognosis. In general, the accuracy of the forecasts improves as MSE approaches 0. Comparing the ASE and RMSE values, they should be somewhat close. There should not be much of a difference between the RMSSE and 1. The predicted variability is underestimated if RMSSE is larger than 1, and it is overestimated if it is less than 1. In Merbil, the highest ME was found in September (−0.556), and the lowest was recorded in March (−0.005). Similarly, the RMSE was also highest in September (20.865) and lowest in March (2.121). The values of MSE in all months were closer to 0, which indicates more prediction accuracy. The values of RMSSE were less than 1 in all months, indicating the variability is overestimated (Table 3).

**Table 3:** Accuracy measurement using cross validation result in ordinary kriging method.

Month	ME	RMSE	MSE	RMSSE	ASE	TE
January	−0.041	5.124	−0.002	0.897	5.780	5.84
February	−0.011	3.215	0.001	0.913	3.568	3.64
March	−0.005	2.121	0.001	0.904	2.377	2.47
April	−0.017	2.648	−0.001	0.881	3.050	3.15
May	−0.077	3.867	−0.011	0.904	4.317	4.32
June	−0.076	6.334	−0.003	0.904	7.063	7.08
July	−0.093	11.227	−0.001	0.914	12.435	12.42
August	−0.448	16.478	−0.017	0.934	17.843	17.44
September	−0.556	20.865	−0.012	0.942	22.384	21.87
October	−0.421	17.312	−0.016	0.941	18.576	18.19
November	−0.233	11.351	−0.009	0.930	12.493	12.32
December	−0.147	6.528	−0.019	0.899	7.389	6.33

### Floristic composition of aquatic plants

Based on the floristic survey, a total of 41 plant species belonging to 23 families were identified in this small freshwater wetland. Table 4 contains a complete species list and their family and habitat. *Cyperaceae* (seven species), *Poaceae* (four), *Hydrocharitaceae*, *Nymphaeaceae,* and *Pontederiaceae* (three species each) had the most species richness. Some of the families, like *Alismataceae, Amaranthaceae, Salviniaceae,* etc., were only one species. Out of the total, 25 species were emergent, 11 were floating leaves, and the remaining five were free-floating habitats.

**Table 4:** List of species with their family and habitat identified in Merbil wetland (alphabetically arranged by families).

Sl no	Species	Common name	Family	Ecological habitat
1	*Sagittaria sagittifolia*	Arrowhead	Alismataceae	Emergent
2	*Alternanthera Philoxeroides*	Alligator Weed	Amaranthaceae	Emergent
3	*Pistia*	Water Lettuce	Araceae	Free Floating
4	*Lemna perpusilla*	Minute duckweed	Araceae	Free Floating
5	*Enydra fluctuans*	–	Asteraceae	Emergent
6	*Brasenia Schreberi*	Water-Shield	Cabombaceae	Floating-leave
7	*Ipomoea Aquatica*	Swamp Morning Glory	Convolvulaceae	Emergent
8	*Ipomoea carnea*	Morning Glowry	Convolvulaceae	Emergent
9	*Scirpoides holoschoenus (L.)*	Roundhead Bulrush	Cyperaceae	Emergent
10	*Scirpus*	Grassweed/ClubRush	Cyperaceae	Emergent
11	*Cyperus digitatus*	Finger Flatsedge	Cyperaceae	Emergent
12	*Eleocharis acutangula*	–	Cyperaceae	Emergent
13	*Eleocharis dulci*	Water chesnut	Cyperaceae	Emergent
14	*Fuirena ciliaris*	–	Cyperaceae	Emergent
15	*Actinoscirpus grossus*	–	Cyperaceae	Emergent
16	*Myriophyllum indicum*	–	Haloragaceae	Emergent
17	*Hydrocharis morsus-ranae*	Common frogbit	Hydrocharitaceae	Free Floating
18	*Ottelia alismoides*	Duck Lettuce	Hydrocharitaceae	Floating-leave
19	*Hydrilla verticillata*	Water Thyme	Hydrocharitaceae	Emergent
20	*Trapa natans*	Water Chestnut	Lythraceae	Floating-leave
21	*Marsilea quadrifolia*	Water Shamrock	Marsileaceae	Floating-leave
22	*Nymphoides indica*	Water Snow Flake	Menyanthaceae	Floating-leave
23	*Nelumbo nucifera*	Indian Lotus	Nelumbonaceae	Floating-leave
24	*Nymphaea nouchali*	Water Lily	Nymphaeaceae	Floating-leave
25	*Euryale ferox*	Prickly Water Lily	Nymphaeaceae	Floating-leave
26	*Nymphaea albea*	White Waterlily	Nymphaeaceae	Floating-leave
27	*Ludwigia octovavis*	Primrose willow	Onagraceae	Emergent
28	*Ludwigia peploides*	Floating Primrose	Onagraceae	Emergent
29	*Bacopa monnieri*	Brahmi	Plantaginaceae	Emergent
30	*Hygroryza aristata*	Asian Water Grass	Poaceae	Floating-leave
31	*Hymenachne assamica*	Marsh grasses	Poaceae	Emergent
32	*Phragmites karka*	Tall Reed	Poaceae	Emergent
33	*Leersia oryzoides*	Rice Cutgrass	Poaceae	Emergent
34	*Monochoria hastata*	Arrow leaf pondweed	Pontederiaceae	Emergent
35	*Pontederia cordata*	Pickerelweed	Pontederiaceae	Emergent
36	*Eichhornia crassipes*	Water Hyacinth	Pontederiaceae	Free Floating
37	*Polygonum glabram*	Common Marsh	Polygonaceae	Emergent
38	*Potamogeton natans*	Pondweed	Potamogetonaceae	Floating-leave
39	*Salvinia natans*	Floating Water moss	Salviniaceae	Free Floating
40	*Cyclosorus interruptus*	Hottentott Fern	Thelypteridaceae	Emergent
41	*Alpinia nigra*	Tara	Zingiberaceae	Emergent

### Density of aquatic plants

Plant density simply refers to the number of individuals per unit area, which has a significant role in increasing the biomass of the plant. In the study site, the plant density and relative density were investigated in the seasons of pre-monsoon, monsoon, post-monsoon, and winter (Table 5). In the pre-monsoon season, *Scirpoides holoschoenus (L.)* was the highest density (14.35 plants/m^2^) and followed by *Hygroryza aristata* (11.36 plants/m^2^), *Eichhornia crassipes* (10.38 plants/m^2^), *Scirpus* (9.55 plants/m^2^), and *Hymenachne assamica* (8.35 plants/m^2^). On the other hand, plants like *Myriophyllum indicum, Sagittariasa gittifolia, Fuirena ciliaris, Enydra fluctuans,* and *Eleocharis dulci* had a density of less than one plant/m^2^. *Eichhornia crassipes* (21.56 plants/m^2^) was recorded as the highest density in the season of monsoon and followed by *Scirpoides holoschoenus (L.)* (10.93 plants/m^2^), *Marsilea quadrifolia* (7.85 plants/m^2^), and *Scirpus* (7.85 plants/m^2^). In this season, some emergent aquatic plants, namely *Eleocharis dulci, Enydra fluctuans, Fuirena ciliaris,* and *Hymenachne assamica,* were totally absent. *Eichhornia crassipes* was again noted in post-monsoon and winter as the most dominant aquatic plant in the wetland, with a plant density of 40.07 plants/m^2^ and 25.91 plants/m^2^, respectively. Other notable aquatic plants in both seasons included *Scirpoides holoschoenus (L.)* and *Hygroryza aristata*.

**Table 5:** Season wise density, relative density, frequency, and relative frequency of the aquatic plants at the wetland.

Sl no	Botanical name	Pre monsoon				Monsoon				Post monsoon				Winter			
		**D**	**RD**	**F**	**RF**	**D**	**RD**	**F**	**RF**	**D**	**RD**	**F**	**RF**	**D**	**RD**	**F**	**RF**
Free Floating																	
*1*	*Eichhornia crassipes*	*10.38*	*6.81*	*63.64*	*7.78*	*21.56*	*17.2*	*76.36*	*13.5*	*40.07*	*32.63*	*78.18*	*17*	*25.91*	*20.59*	*74.55*	*15.3*
*2*	*Pistia*	*4.33*	*2.84*	*30.91*	*3.78*	*5.47*	*4.36*	*34.55*	*6.11*	*5.85*	*4.77*	*34.55*	*7.51*	*4.53*	*3.6*	*27.27*	*5.6*
*3*	*Salvinia natans*	*6.51*	*4.27*	*34.55*	*4.22*	*5.45*	*4.35*	*27.27*	*4.82*	*3.45*	*2.81*	*16.36*	*3.56*	*3.64*	*2.89*	*16.36*	*3.36*
*4*	*Hydrocharis morsus-ranae*	*5.89*	*3.87*	*49.09*	*6*	*3.44*	*2.74*	*23.64*	*4.18*	*2.82*	*2.29*	*20*	*4.35*	*2.67*	*2.12*	*14.55*	*2.99*
*5*	*Lemna perpusilla*	*4.91*	*3.22*	*9.09*	*1.11*	*4.85*	*3.87*	*9.09*	*1.61*	*2.38*	*1.94*	*3.64*	*0.79*	*2.6*	*2.07*	*3.64*	*0.75*
Floating Leaves																	
*6*	*Nymphaea albea*	*0.98*	*0.64*	*16.36*	*2*	*0.84*	*0.67*	*10.91*	*1.93*	*1.11*	*0.9*	*12.73*	*2.77*	*1.33*	*1.05*	*14.55*	*2.99*
*7*	*Ottelia alismoides*	*1.15*	*0.75*	*12.73*	*1.56*	*1.11*	*0.88*	*14.55*	*2.57*	*1.07*	*0.87*	*14.55*	*3.16*	*1.45*	*1.16*	*14.55*	*2.99*
*8*	*Hygroryza aristata*	*11.36*	*7.46*	*41.82*	*5.11*	*7.49*	*5.97*	*29.09*	*5.14*	*7.2*	*5.86*	*25.45*	*5.53*	*5.84*	*4.64*	*20*	*4.1*
*9*	*Nelumbo nucifera*	*0.49*	*0.32*	*14.55*	*1.78*	*0.42*	*0.33*	*12.73*	*2.25*	*0.35*	*0.28*	*10.91*	*2.37*	*0.38*	*0.3*	*10.91*	*2.24*
*10*	*Nymphoides indica*	*1.15*	*0.75*	*16.36*	*2*	*1.25*	*1*	*12.73*	*2.25*	*0.98*	*0.8*	*12.73*	*2.77*	*0.82*	*0.65*	*10.91*	*2.24*
*11*	*Nymphaeaceae*	*1.75*	*1.15*	*21.82*	*2.67*	*1.04*	*0.83*	*16.36*	*2.89*	*1.2*	*0.98*	*18.18*	*3.95*	*1.33*	*1.05*	*18.18*	*3.73*
*12*	*Braseniaschreberi*	*1*	*0.66*	*20*	*2.44*	*1.09*	*0.87*	*18.18*	*3.22*	*0.93*	*0.75*	*12.73*	*2.77*	*0.58*	*0.46*	*7.27*	*1.49*
*13*	*Euryale ferox*	*0.55*	*0.36*	*16.36*	*2*	*0.44*	*0.35*	*14.55*	*2.57*	*0.4*	*0.33*	*9.09*	*1.98*	*0.47*	*0.38*	*9.09*	*1.87*
*14*	*Trapa natans*	*8.42*	*5.52*	*45.45*	*5.56*	*5.45*	*4.35*	*30.91*	*5.47*	*3.8*	*3.09*	*23.64*	*5.14*	*3.65*	*2.9*	*20*	*4.1*
*15*	*Potamogeton natans*	*4.8*	*3.15*	*20*	*2.44*	*0.44*	*0.35*	*7.27*	*1.29*	*0.49*	*0.4*	*7.27*	*1.58*	*0.55*	*0.43*	*7.27*	*1.49*
*16*	*Marsilea quadrifolia*	*11*	*7.22*	*20*	*2.44*	*7.85*	*6.26*	*14.55*	*2.57*	*4.24*	*3.45*	*10.91*	*2.37*	*4.65*	*3.7*	*9.09*	*1.87*
Emergent																	
*17*	*Scirpoides holoschoenus (L.)*	*14.35*	*9.41*	*41.82*	*5.11*	*10.93*	*8.72*	*16.36*	*2.89*	*9.07*	*7.39*	*9.09*	*1.98*	*9.29*	*7.38*	*12.73*	*2.61*
*18*	*Scirpus*	*9.55*	*6.26*	*27.27*	*3.33*	*7.85*	*6.26*	*14.55*	*2.57*	*7.87*	*6.41*	*10.91*	*2.37*	*8.89*	*7.07*	*14.55*	*2.99*
*19*	*Cyclosorus interruptus*	*6.22*	*4.08*	*36.36*	*4.44*	*5.65*	*4.51*	*20*	*3.54*	*0.82*	*0.67*	*5.45*	*1.19*	*5.42*	*4.31*	*12.73*	*2.61*
*20*	*Pontederia cordata*	*1.65*	*1.09*	*23.64*	*2.89*	*0.56*	*0.45*	*9.09*	*1.61*	*0.89*	*0.73*	*9.09*	*1.98*	*1.2*	*0.95*	*10.91*	*2.24*
*21*	*Ludwigia octovavis*	*1.8*	*1.18*	*20*	*2.44*	*1.42*	*1.13*	*16.36*	*2.89*	*1.2*	*0.98*	*10.91*	*2.37*	*1.04*	*0.82*	*9.09*	*1.87*
*22*	*Hymenachne assamica*	*8.35*	*5.48*	*30.91*	*3.78*	*0*	*0*	*0*	*0*	*0*	*0*	*0*	*0*	*3.04*	*2.41*	*7.27*	*1.49*
*23*	*Phragmites karka*	*0.87*	*0.57*	*7.27*	*0.89*	*1.04*	*0.83*	*7.27*	*1.29*	*1.11*	*0.9*	*7.27*	*1.58*	*1*	*0.79*	*7.27*	*1.49*
*24*	*Polygonum glabram*	*4.6*	*3.02*	*41.82*	*5.11*	*3.09*	*2.47*	*14.55*	*2.57*	*0*	*0*	*0*	*0*	*0.42*	*0.33*	*5.45*	*1.12*
*25*	*Ipomoea aquatica*	*0.91*	*0.6*	*9.09*	*1.11*	*1.2*	*0.96*	*10.91*	*1.93*	*0.75*	*0.61*	*7.27*	*1.58*	*0.82*	*0.65*	*7.27*	*1.49*
*26*	*Alternantherap hiloxeroides*	*2.07*	*1.36*	*10.91*	*1.33*	*1.98*	*1.58*	*9.09*	*1.61*	*1.4*	*1.14*	*5.45*	*1.19*	*1.49*	*1.18*	*7.27*	*1.49*
*27*	*Ludwigia peploides*	*2.4*	*1.57*	*20*	*2.44*	*2.64*	*2.1*	*20*	*3.54*	*2.04*	*1.66*	*12.73*	*2.77*	*2.45*	*1.95*	*14.55*	*2.99*
*28*	*Leersia oryzoides*	*2.8*	*1.84*	*12.73*	*1.56*	*2.71*	*2.16*	*7.27*	*1.29*	*2.24*	*1.82*	*3.64*	*0.79*	*4.84*	*3.84*	*9.09*	*1.87*
*29*	*Actinoscirpus grossus*	*4.45*	*2.92*	*12.73*	*1.56*	*3.25*	*2.6*	*9.09*	*1.61*	*3.42*	*2.78*	*7.27*	*1.58*	*3.51*	*2.79*	*7.27*	*1.49*
*30*	*Alpinia nigra*	*0.49*	*0.32*	*5.45*	*0.67*	*0.6*	*0.48*	*5.45*	*0.96*	*0.56*	*0.46*	*5.45*	*1.19*	*0.51*	*0.4*	*5.45*	*1.12*
*31*	*Bacopa monnieri*	*1.6*	*1.05*	*7.27*	*0.89*	*0.35*	*0.28*	*1.82*	*0.32*	*0.33*	*0.27*	*1.82*	*0.4*	*0.71*	*0.56*	*5.45*	*1.12*
*32*	*Cyperus digitatus*	*3.56*	*2.34*	*3.64*	*0.44*	*4.38*	*3.49*	*5.45*	*0.96*	*3.84*	*3.12*	*3.64*	*0.79*	*6.51*	*5.17*	*9.09*	*1.87*
*33*	*Eleocharis acutangula*	*4.42*	*2.9*	*5.45*	*0.67*	*3.45*	*2.76*	*3.64*	*0.64*	*3.65*	*2.98*	*3.64*	*0.79*	*4.75*	*3.77*	*7.27*	*1.49*
*34*	*Eleocharis dulci*	*0.65*	*0.43*	*7.27*	*0.89*	*0*	*0*	*0*	*0*	*0*	*0*	*0*	*0*	*0.47*	*0.38*	*3.64*	*0.75*
*35*	*Enydra fluctuans*	*0.55*	*0.36*	*3.64*	*0.44*	*0*	*0*	*0*	*0*	*0*	*0*	*0*	*0*	*0.22*	*0.17*	*1.82*	*0.37*
*36*	*Fuirena ciliaris*	*0.16*	*0.11*	*1.82*	*0.22*	*0*	*0*	*0*	*0*	*0*	*0*	*0*	*0*	*0.65*	*0.52*	*3.64*	*0.75*
*37*	*Hydrilla verticillata*	*2.78*	*1.83*	*16.36*	*2*	*2.22*	*1.77*	*10.91*	*1.93*	*1.84*	*1.5*	*9.09*	*1.98*	*2.78*	*2.21*	*10.91*	*2.24*
*38*	*Ipomoea carnea*	*1.31*	*0.86*	*14.55*	*1.78*	*1.2*	*0.96*	*9.09*	*1.61*	*1.33*	*1.08*	*9.09*	*1.98*	*1.24*	*0.98*	*5.45*	*1.12*
*39*	*Monochoria hastata*	*1.15*	*0.75*	*16.36*	*2*	*1.62*	*1.29*	*12.73*	*2.25*	*2.4*	*1.95*	*16.36*	*3.56*	*2.33*	*1.85*	*16.36*	*3.36*
*40*	*Myriophyllum indicum*	*0.71*	*0.47*	*5.45*	*0.67*	*0.42*	*0.33*	*3.64*	*0.64*	*0.71*	*0.58*	*3.64*	*0.79*	*0.73*	*0.58*	*5.45*	*1.12*
*41*	*Sagittariasa gittifolia*	*0.33*	*0.21*	*3.64*	*0.44*	*0.62*	*0.49*	*5.45*	*0.96*	*1.02*	*0.83*	*7.27*	*1.58*	*1.15*	*0.91*	*9.09*	*1.87*

**Notes.**

D density RD relative density F frequency RF relative frequency

*Eichhornia crassipes* had the highest density in the Merbil wetland except for pre-monsoon. Since the wetland’s water level was relatively lower during the dry season compared to other seasons, most of the water hyacinth-covered portions were dried out and decomposed. After reducing the area occupied by *Eichhornia crassipes*, many emergent plant species, including *Hymenachne assamica, Eleocharis acutangula, Actinoscirpus grossus, Enydra fluctuans*, etc., were grown in the wetland. Moreover, the number of floating-leaved plants increased during this season. In the monsoon and post-monsoon season, the density of *Eichhornia crassipes* risen rapidly with the increase in water level, rainfall, and temperature. It occupied most of the wetland area in these seasons, and accordingly, the density of other plants in the wetland decreased rapidly, especially emergent plants. Although the *Eichhornia crassipes* had the highest density in the winter season, as a result of the lower rainfall and temperature, they had started to decline from the previous seasons. In contrast, the density of emergent plants progressively increased this season.

### Frequency of aquatic plants

Frequency is the number of times a species occurs in a given sample point (Sewale & Mammo, 2022) or the percentage of sample points in which a species of interest occurs (Cox, Booth & Berryman, 2021). Frequency measures the presence or absence of a species within the area of interest and is often used to compare plant communities and detect changes in floristic composition over time. The higher the frequency, the more important the plant is in the community. In the study site, the most frequent species in pre-monsoon was *Eichhornia crassipes* (frequency and relative frequencies were 63.64 and 7.78, respectively), followed by *Hydrocharis morsus-ranae* (frequency and relative frequency were 49.09 and 6, respectively), *Trapa natans* (frequency and relative frequency 45.45 and 5.56 respectively), *Polygonum glabram* (frequency and relative frequency were41.82 and 5.11 respectively), *Scirpoides holoschoenus* (frequency and relative frequency were41.82 and 5.11 respectively) and so on. Plants like *Phragmites karka*, *Alpinianigra, Bacopa monnieri, Cyperus digitatus, Eleocharis acutangula, Eleocharis dulci, Enydra fluctuans, Fuirena ciliaris, Myriophyllum indicum,* and *Sagittariasa gittifolia* had the frequency and relative frequency of <10 and <1 during this month respectively. In the season of monsoon, *Eichhornia crassipes* (frequency of 76.36 and relative frequency of 13.5) again observed as the most frequent plant in the wetland. It was followed by *Pistia* (frequency and relative frequency were 34.55 and 6.11 respectively), *Trapa natans* (frequency and relative frequency were 30.91 and 5.47 respectively) *Hygroryza aristata* (frequency and relative frequency were 29.09 and 5.14 respectively), *Salvinia natans* (frequency and relative frequency were 27.27 and 4.82 respectively) etc. Frequency of many emergent plants namely *Hymenachne assamica, Eleocharis dulci, Enydra fluctuans,* and *Fuirena ciliaris* became nil in this season.

In post-monsoon, the frequency and relative frequency of *Eichhornia crassipess* lightly increased and reached 78.18 and *17* respectively. The other most frequent plants in the study area during post-monsoon were *Pistia* (frequency and relative frequencies were 34.55 and 7.51, respectively), *Hygroryza aristata* (frequency and relative frequency were 25.45 and 5.53 respectively), *Trapa natans* (frequency and relative frequency were 23.64 and 5.14 respectively), and *Hydrocharis morsus-ranae* (frequency and relative frequency were 20 and 4.35 respectively). *Polygonum glabram, Eleocharis dulci, Enydra fluctuans, Fuirena ciliaris,* and *Hymenachne assamica* were among the plants whose frequency was reported to be zero. In winter season, again *Eichhornia crassipeshad* reported as highest (frequency and relative frequency were 74.55 and 5.3 respectively) frequent aquatic plants out of the total. Other frequent species were *Pistia* (frequency and relative frequency 27.27 and 5.6), *Salvinia natans* (frequency and relative frequency were 16.36 and 3.36, respectively), and *Nymphaeaceae* (frequency and relative frequency were 18.18 and 3.73, respectively). *Lemna perpusilla, Eleocharis dulci, Enydra fluctuans, and Fuirena ciliaris* were reported as less frequent species with a frequency of <5.

According to the observations, *Eichhornia crassipes* was the wetland’s most dominant plant. It was reported as the most frequent plant in all four study months and found in the Merbil wetland throughout the year. However, its density and frequency had changed with the environmental conditions. In monsoon and post-monsoon, the frequency of *Eichhornia crassipes* was >75 due to ideal growing conditions. The frequency of maximum emergent plants was observed in the pre-monsoon and winter seasons. The frequency of floating-leaved plants also fluctuates with the changes in surrounding environmental conditions.

### Estimation of species diversity of aquatic plants

The Shannon-Wiener diversity indices (*H*′), species richness, Shannon’s equitability or evenness (J), Simpson diversity index, and dominance (D) were used to assess the species diversity of aquatic plants in Merbil wetland. The highest species richness was found in the season of pre-monsoon and winter (41 species in both) and followed by monsoon (37) and post-monsoon (36) (Table 6). Many emergent plants were grown in the pre-monsoon as well as the winter season due to the decrease in the water surface of the wetland. Thus, the species richness of pre-monsoon and winter seasons was high compared to the monsoon and post-monsoon seasons.

**Table 6:** Species diversity parameters in different seasons.

Season	No. of species	Dominance	Shannon (*H*′)	Simpson	Evenness
Pre-monsoon	41	0.047	3.29	0.95	0.65
Monsoon	37	0.064	3.12	0.92	0.61
Post-monsoon	36	0.130	2.97	0.87	0.45
Winter	41	0.071	3.14	0.93	0.56

**Figure 7:**
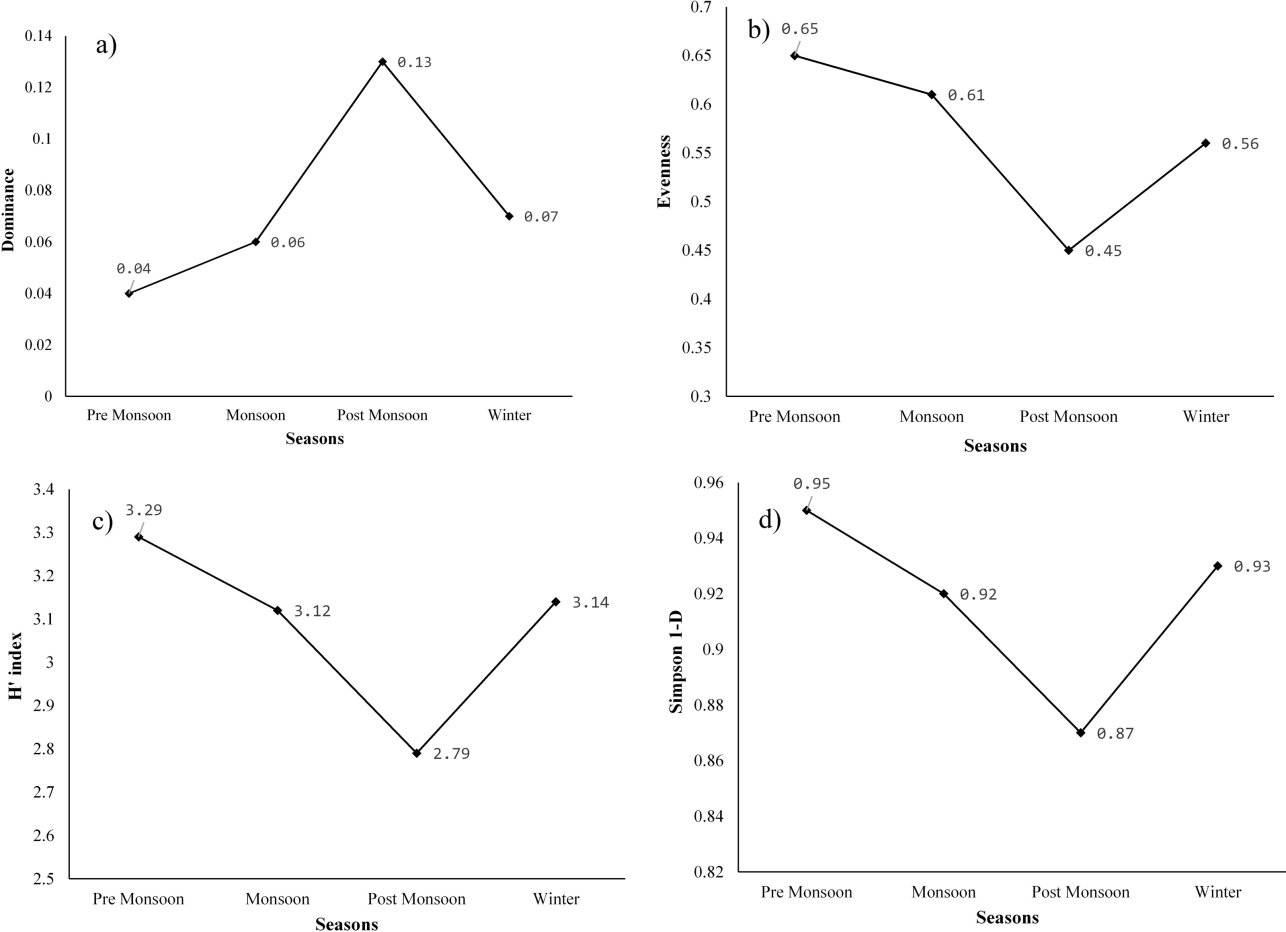
(A) Dominance of plants, (B) equitability or evenness of plants, (C) Shannon-Weiner diversity index (*H*), and (D) Simpson diversity index of species of the study site.

The highest dominance (D) of species was recorded in the post-monsoon season (0.13), when water hyacinth covered the majority of the wetland area and followed by winter (0.07), monsoon (0.06), and pre-monsoon (0.04). After the post-monsoon season, other aquatic plants, especially emergent plants, grew denser, leading to a steady decline in water hyacinth dominance. Dominance (D) generally ranges from 0 (all taxa are equally present) to 1 (one taxon dominates the community completely). Evenness or equitability assumes a value between 0 and 1. The higher the value of the evenness index, the more even the species is in their distribution within the given area. The highest evenness was observed in the pre-monsoon season (0.65), and the lowest was recorded in the post-monsoon season (0.45). The evenness of the monsoon and winter seasons was 0.61 and 0.56, respectively (Fig. 7). The post-monsoon season experienced low evenness due to the high dominance of a few aquatic plants. In other words, the plant species were not evenly distributed during this period. However, it was distributed more evenly in the pre-monsoon season than in other seasons.

The highest Shannon (*H*′) index was found in the pre-monsoon season (3.29) and was followed by winter (3.14), monsoon (3.12), and post-monsoon season (2.97). Similarly, the maximum Simpson diversity was also observed in the season of pre-monsoon (0.95), and it was followed by winter (0.93), monsoon (0.92), and post-monsoon (0.87). In the pre-monsoon season, both Shannon and Simpson diversity was highest in the wetland due to the high evenness as well as low dominance of the species. Moreover, the species richness was also high during this season. In contrast, the post-monsoon season had very high dominance and a decline in evenness to 0.45, which resulted in low species diversity at the study site. As per the field survey data, out of the total individuals, 32.68 percent were only water hyacinth in the post-monsoon season. After the post-monsoon, species diversity began to increase again, with a decrease in dominance and an increase in evenness.

## Discussion

The highly reproductive nature of water hyacinth poses a serious threat to biodiversity since it readily outcompetes other species (Ayanda, Ajayi & Asuwaju, 2020). The study’s result showed that the wetland’s water hyacinth biomass affected the floristic composition of aquatic plants . After pre-monsoon, water hyacinth density and biomass increased very rapidly in the Merbil wetland, reducing the density and frequency of other aquatic plants and species diversity of the wetland. In the pre-monsoon season, the water hyacinth density was 10.37 plants/m^2^ and increased to 21.56 plants/m^2^ and 40.07 plants/m^2^ in the monsoon and post-monsoon seasons, respectively. However, during winter, it decreased and became 25.91 plants/m^2^. Similarly, the frequency of water hyacinth was also changed in the same pattern as density. As shown in Table 5, the increasing density and frequency of water hyacinths in the different seasons alter the density and frequency of the other aquatic plants. Even though some species of aquatic plants from the Poaceae and Cyperaceae families seem to coexist with water hyacinth, this invasive species has decreased plants density, frequency, and diversity in the infested months and, in some cases, converted the community to nearly monotypic flora.

Table 7 shows the correlation of water hyacinth biomass with the Shannon (*H*′) index, Simpson diversity index, and evenness. According to the study, the Simpson diversity and Shannon (*H*′) indices were low during the peak infestation season of the water hyacinth and high during the low infestation season. Additionally, species’ evenness during the infested season was less than what it was during the non-infested season. Thus, a negative correlation was observed between water hyacinth biomass and the Shannon (*H*′) index, Simpson diversity index, and evenness. It indicates the drastic impact of water hyacinth biomass on plant species diversity. Moreover, the dominance of plants had a positive correlation with water hyacinth biomass. It was due to the increase of dominance with the growth of water hyacinth infestation and biomass. The result shows that water hyacinth has the potential to alter the floristic composition of aquatic plants in freshwater ecosystems. Similar results were found by Mengistu, Unbushe & Abebe (2017) in Lake Abaya, Ethiopia, where the invasive water hyacinth affects the composition of macrophytes, their abundance, and species diversity. Ayanda, Ajayi & Asuwaju (2020) studied the threat of water hyacinth on water bodies and stated that the fast reproductive nature of water hyacinth poses a great threat to biodiversity as the weed easily outcompetes other species.

**Table 7:** Pearson correlation coefficient between water hyacinth biomass and diversity indices.

**Diversity indices**	***r* value**	***p* value**
Shannon (*H*′)	−0.92	0.07
Simpson	−0.97	0.02
Dominance	0.99	0.001
Evenness	−0.97	0.02

In the present study, the results indicated that the change in water hyacinth biomass significantly affects the floristic composition and species diversity of the aquatic weeds. Like merbil, the Amazon basin in South America, many freshwater aquatic bodies in East and South East Asia, Lake Tana and Lake Victoria in Africa, water bodies covering nearly half of China’s territory (Lu et al., 2007), and many other freshwater lakes around the world especially in the tropical and subtropical region are all being negatively impacted by water hyacinth biomass. Thus, this kind of research is very relevant in other parts of the world where a thick mat of water hyacinth cover has infested freshwater aquatic bodies. The study will aid in developing natural resource sustainability management plans and provide useful guidelines for preserving the local ecological balance in small wetlands over the short to medium term.

There are a number of limitations in the methodology for estimating water hyacinth biomass, which we have adapted here. For example, we used the direct biomass sampling method because it was simple to calculate and had a lower error rate. Still, this method prevents continuous monitoring of the same plant stands and may add spatial variability because it involves sampling a different plot each time. We choose a small study area since collecting samples in an aquatic environment is expensive, time-consuming, and dangerous. It would be more effective if further research applied this method to large bodies of water. Furthermore, the remarkable amount of living plant mass makes it particularly challenging to gather the data required to calculate plant biomass and productivity reliably.

## Conclusions

According to the results of the present study, water hyacinth generates a huge quantity of biomass and has a significant impact on the floristic characteristics of aquatic plants, such as density, frequency, diversity, evenness, and dominance. Although some species seem to coexist with the water hyacinth, most plant species were adversely affected by the growth of water hyacinth biomass. The invasion of water hyacinth could be restricted by continuous monitoring at the controllable stage. Effective control techniques have been found, however, they need to be scaled up for best utility. This invasive plant has a lot of prospects also. Water hyacinth is a promising substrate for bio-ethanol production due to its high carbohydrate content and ready availability (Ganguly, Chatterjee & Dey, 2012; Rezania et al., 2015). Dry water hyacinth biomass is rich in cellulose (18–31 percent) and hemicellulose (18–43 percent) but low in lignin (7–26 percent), making it a good candidate for hydrolyzing to reduce sugars and subsequently fermenting to bio-ethanol using efficient yeasts. Water hyacinth can be used as a biofertilizer due to its high nitrogen (N), phosphorous (P), and potassium (K) content (on a dry basis, of course). There is also a lot of room for innovation in the handicraft industry using this material. The dried plant and its fiber can be used to create a wide variety of useful goods, including bags, handbags, wallets, flower pots, fashion accessories, mats, and many other things. Numerous pilot plant and laboratory tests are being done all around the world, with potentially exciting results. It’s time we stopped thinking of this common weed as a nuisance and started seeing it for what it really is: a resource with vast, untapped value. Weed is a resource with unrealized potential for broad benefits rather than a nuisance.

##  Supplemental Information

10.7717/peerj.14811/supp-1 Supplemental Information 1 Raw data for Table 1, 3 and Figure 4

10.7717/peerj.14811/supp-2 Supplemental Information 2 Raw data for Table 2 and Figure 5, 6

10.7717/peerj.14811/supp-3 Supplemental Information 3 Raw data for Table 5

10.7717/peerj.14811/supp-4 Supplemental Information 4 Raw data for Table 6 and Figure 7

10.7717/peerj.14811/supp-5 Supplemental Information 5 Raw data for Table 7

## Additional Information and Declarations

### Data Availability

The following information was supplied regarding data availability: The raw data is available in the Supplemental Files.
